# Depression and cognition are associated with lipid dysregulation in both a multigenerational study of depression and the National Health and Nutrition Examination Survey

**DOI:** 10.1038/s41398-024-02847-6

**Published:** 2024-03-12

**Authors:** S. M. A. Mehdi, A. P. Costa, C. Svob, L. Pan, W. J. Dartora, A. Talati, M. J. Gameroff, P. J. Wickramaratne, M. M. Weissman, L. B. J. McIntire

**Affiliations:** 1 Mailman School of Public Health, Columbia University, New York, NY USA; 2 Department of Radiology, Weill Cornell Medicine, New York, NY USA; 3 Brain Health Imaging Institute, New York, NY USA; 4 Division of Translational Epidemiology and Mental Health Equity, New York State Psychiatric Institute, New York, NY USA; 5 Department of Psychiatry, Columbia University, New York, NY USA; 6 Present Address: Department of Pathology and Cell Biology, Columbia University, New York, NY USA

**Keywords:** Learning and memory, Depression

## Abstract

Chronic dysregulation of peripheral lipids has been found to be associated with depression and cognition, but their interaction has not been investigated. Growing evidence has highlighted the association between peripheral lipoprotein levels with depression and cognition with inconsistent results. We assessed the association between peripheral lipids, depression, and cognition while evaluating their potential interactions using robust clinically relevant predictors such as lipoprotein levels and chronic medical disorders that dysregulate lipoproteins. We report an association between peripheral lipids, depression, and cognition, suggesting a common underlying biological mechanism driven by lipid dysregulation in two independent studies. Analysis of a longitudinal study of a cohort at high or low familial risk for major depressive disorder (MDD) (*n* = 526) found metabolic diseases, including diabetes, hypertension, and other cardiovascular diseases, were associated with MDD and cognitive outcomes. Investigating a cross-sectional population survey of adults in the National Health and Nutrition Examination Survey 2011–2014 (NHANES) (*n* = 2377), depression was found to be associated with high density lipoprotein (HDL) and cognitive assessments. In the familial risk study, medical conditions were found to be associated with chronic lipid dysregulation and were significantly associated with MDD using the structural equation model. A positive association between chronic lipid dysregulation and cognitive scores was found in an exploratory analysis of the familial risk study. In a complementary study, analysis of NHANES revealed a positive association of HDL levels with cognition. Further analysis of the NHANES cohort indicated that depression status mediated the interaction between HDL levels and cognitive tests. Importantly, the protective effect of HDL on cognition was absent in those with depressive symptoms, which may ultimately result in worse outcomes leading to cognitive decline. These findings highlight the potential for the early predictive value of medical conditions with chronic lipid dyshomeostasis for the risk of depression and cognitive decline.

## Introduction

Multiple studies have found that peripheral lipoproteins, which are associated with chronic medical conditions, including cardiovascular disease, are linked to depression and cognitive decline. Lipid parameters exert their influence on cognition not only through direct biological pathways but may also affect it indirectly through psychological factors like depression. Depression itself is known to be a major risk factor for neurocognitive decline [1]. Considering the link between lipoproteins and depression, we hypothesized that the presence of depression plays a mediating role in the association between lipoprotein levels and cognition. Few studies have attempted to investigate this interaction, however, multiple studies have identified a strong association between atherogenic factors and depression. For example, decreased total serum cholesterol, decrease in high-density lipoprotein (HDL), and increase in low-density lipoprotein (LDL) and LDL/HDL ratio, are associated with depression [2–9]. A study by the National Health and Nutrition Examination Survey (NHANES; 2015–2016) showed that the level of TG was one of the most important features in predicting depression [10]. However, conflicting results from previous analyses of NHANES (2009–2015) reported that low levels of total cholesterol (TC) were not associated with an increased risk of depression [11], while two other studies reported an inverse relationship between depression and levels of TC [12–14]. Recently, Jia et al. [15] showed that HDL was positively correlated with depressive symptom severity, but LDL, triglyceride levels (TG), and TC were negatively correlated with depressive symptom severity as well as cognitive performance [15]. More recent analyses of the NHANES have reported that non-high-density lipoprotein cholesterol, an increase in atherogenic coefficient, or an increase in the non-high-density lipoprotein cholesterol to high-density lipoprotein cholesterol ratio were associated with an increased prevalence of depression [16–18].

Chronic dysregulation of peripheral lipids, including HDL, LDL, TC, and TG levels, has been shown to predict cognitive performance [19–21], suggesting potential shared mechanisms determining pathological outcomes. Lipid dyshomeostasis can affect cognition through direct biological pathways such as synaptic transmission, neuroplasticity, and antidepressant action [22–26]. Further, depression itself is known to be a major risk factor for neurocognitive decline [1]. However, studies to date have shown conflicting outcomes and have been limited by sample size. It is, therefore, not known if chronic lipid dyshomeostasis shares a biological mechanism with underlying neurological changes leading to changes in depressive state and/or cognition. Given the association of lipid dysregulation with cognitive deficits [19–21], and major depressive disorder (MDD), a biological pathway involving lipid dyshomeostasis is highly likely. For example, a previous study in unipolar and bipolar depression found cognitive abilities were associated with altered levels of Apolipoprotein B (ApoB) [27]. Another study found TG levels significantly correlated with cognition in those with MDD compared to healthy controls [28]. Further, in NHANES (cohort 2011–2014), depression was associated with decreased cognitive scores and shown to be synergistic with diabetes, thus highlighting the impact of additional metabolic disorders on cholesterol pathways [29]. Considering the link between lipoproteins and depression, we posit that the presence of depression plays a mediating role in the association between lipoprotein levels and cognition.

Multiple chronic medical conditions and metabolic diseases are known to disrupt lipid metabolism over the lifetime including diabetes, gallbladder disease, hypertension, and cardiovascular disease [30–32]. In this study, we aimed to elucidate the relationship between lipid dyshomeostasis, depression, and cognition. Our study tested the same biological hypothesis in two independent and distinct populations, a deeply phenotyped 30-year cohort study of individuals at high and low risk for depression based on family history, and a highly powered study, the National Health and Nutrition Examination Survey (NHANES). We addressed three hypotheses (1) medical conditions such as diabetes, gallbladder disease, hypertension, and other cardiovascular diseases, which reflect chronic dysfunction in lipid metabolism, are likely to be associated with lifetime MDD in a longitudinal study of familial risk for depression; (2) these lipid-associated medical disorders (LAMD) are associated with decreased cognitive performance in the familial risk study; and (3) based on our biological hypothesis, we analyzed a highly powered cross-sectional study, the NHANES, using different methodologies to test the association and potential interactions among depression, lipids, and cognition. The NHANES study, which has more robust and clinically significant predictors such as clinical measures of peripheral lipids, including HDL, was analyzed to test the hypothesis that depression mediates the interaction of peripheral lipids and cognition.

## Methods

### Familial risk study analysis

#### Population

The analysis is based on a cohort of individuals at high and low risk for depression based on family history (*n* = 526). In the original familial risk study, individuals (first generation=G1) with MDD were recruited from New Haven, Connecticut. Nondepressed probands were selected, at the same time, from an epidemiologic sample of adults in the same area and had no history of psychiatric illness, as determined by multiple interviews. For generations 2 (G2) and 3 (G3), high risk was defined as having at least one parent or grandparent, respectively, diagnosed with MDD. The New York State Psychiatric Institute IRB approved all procedures, and informed consent was obtained. The study began in 1982, and there were seven waves (W1-W7) of data collection, which occurred at baseline, and 2, 10, 20, 25, 30, and 35 years thereafter [33–37]. Demographic characteristics of this study are shown in Table 1. Full details regarding familial risk studies have previously been reported [33–37].

**Table 1:** Demographic characteristics and cumulative rates of medical disorders for main sample and subset.

Characteristic	Main Sample (*N* = 526)			Subset^a^ (*N* = 150)		
	Mean (SD)		Range	Mean (SD)		Range
Age at last interview/W6	32.5 (16.1)		4.4–67.9	31.3 (15.2)		6.0–59.1
	***N*** **(%)**			***N*** **(%)**		
Generation (G)						
G2	273 (51.9)			63 (42.0)		
G3	253 (48.1)			87 (58.0)		
Sex						
Female	279 (53.0)			78 (52.0)		
Male	247 (47.0)			72 (48.0)		
Familial Risk Status						
High Risk	354 (67.3)			90 (60.0)		
Low Risk	172 (32.7)			60 (40.0)		
Lifetime MDD						
Yes	217 (41.3)			78 (52.0)		
No	309 (58.7)			72 (48.0)		
Medical Disorder	**Yes**	**No**	**Unknown**	**Yes**	**No**	
Lipid-Associated Medical Disorder (LAMD)	139 (26.4)	387 (73.6)	0	52 (34.7)	98 (65.3)	
Hypertension	70 (13.3)	434 (82.5)	22 (4.2)	25 (16.7)	125 (83.3)	
Cardiovascular	42 (8.0)	479 (91.1)	5 (0.9)	13 (8.7)	137 (91.3)	
Hyperthyroidism	12 (2.3)	514 (97.7)	0	4 (2.7)	146 (97.3)	
Hypothyroidism	28 (5.3)	480 (91.3)	18 (3.4)	13 (8.7)	137 (91.3)	
Gallbladder disease	18 (3.4)	477 (90.7)	31 (5.9)	8 (5.3)	142 (94.7)	
Diabetes	21 (4.0)	487 (92.6)	18 (3.4)	8 (5.3)	142 (94.7)	
Lipid Independent Medical Disorder (LIMD) (cancer)	37 (7.0)	489 (93.0)	0	15 (10.0)	135 (90.0)	
Intermediately Associated Medical Disorder (IAMD)	108 (20.5)	418 (79.5)	0	29 (19.3)	121 (80.7)	
Convulsion	19 (3.6)	507 (96.4)	0	3 (2.0)	147 (98.0)	
Head injury	57 (10.8)	469 (89.2)	0	22 (14.7)	128 (85.3)	
Stroke	41 (7.8)	485 (92.2)	0	7 (4.7)	143 (95.3)	

^a^The subjects who had records of *Similarities subtest T-scores and Verbal IQ sum of T-scores* at W6.

#### Familial risk study assessments

##### Psychiatric assessments

The assessments used to measure psychiatric symptoms, diagnoses, and general medical problems were previously described [38]. The diagnostic interview used at every wave was the Schedule for Affective Disorders and Schizophrenia–Lifetime Version (SADS-L) [39] for adults and the child version Schedule for Affective Disorders and Schizophrenia for School-Age Children (Kiddie-SADS-E) [40]. Data from all waves were pooled to create one variable indicating a lifetime diagnosis of MDD.

##### General medical problems

A standard medical checklist, which includes 57 conditions [38], was used to collect data on medical illness. Individuals were asked whether a doctor diagnosed them with a particular condition and if any medication was prescribed. Information was collected at each wave, and in the case of minors, parents reported on their children. Medical problems were categorized a priori into 14 distinct categories, representing the bodily site or system affected, and data were combined to create variables indicating a lifetime history of a medical condition.

##### Cognition

A subset of the main sample for cognition scores consisted of 150 individuals from the offspring of the proband generation (G1), including the second generation (G2 = 63) and the third generation (G3 = 87) of the multigenerational study who were interviewed at wave 6 (W6) follow-up (Table 1). The cognitive testing included a detailed battery of assessor-administered criterion-standard tests of speed, reasoning/intelligence, attention, executive function, and memory. Full details about the assessments were previously described including the Wechsler Abbreviated Scale of Intelligence (WASI) [41].

##### Familial risk status

The covariate 'familial risk status' represents the risk status of each individual in the families of generations 2 and 3 of the G1 depressed probands. We used this to indicate whether a relative was at high or low risk for depression based on whether the participant was a high or low-risk offspring based on their relationship with the proband.

#### Cluster analysis

**Fig. 1:**
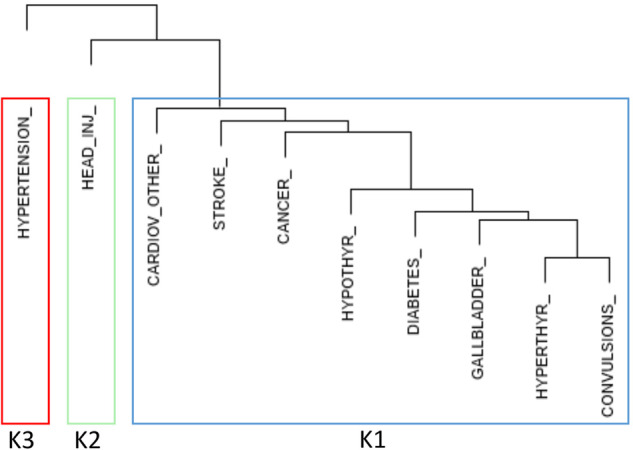
Familial risk study medical disorders cluster into three groups. Shortest Euclidean distance cluster analysis of familial risk cohort with dendrogram (G2 + G3, *N* = 526). An unsupervised algorithm was used to group medical disorders with similar incidence into clusters based on their association strength. To maintain consistency with our primary analysis of LAMD, LIMD, and IAMD, three theoretical groups, *k* = 3 were selected for this analysis.

In an exploratory study, we applied an unsupervised algorithm—hierarchical clustering method-to group medical disorders with similar incidence into clusters based on their closeness (i.e., Euclidean distance) in the R software (version 3.6.1) using package “lavaan”. To maintain consistency with our primary analysis of LAMD, LIMD, and IAMD, three theoretical groups (*k* = 3) were selected for this analysis (Table 2) According to the output of the identified three clusters, we conducted two structural equations models that used *k*1, *k*2, and *k*3 as latent variables to examine their association with lifetime MDD or cognition scores in a parsimonious model and controlled for the potential confounding covariates age, sex, and familial risk status as described above (Fig. 1).

**Table 2:** Structural equation models based on the hierarchical clustering method (Euclidean distance) with lifetime MDD as outcome.

Predictor	Estimate	*z*-value	*P*(>|*z*|)
1. *Basic model*			
*k*1	**2.56**	**2.91**	**0.004**
*k*2	**0.17**	**2.22**	**0.026**
*k*3	0.03	0.30	0.763
2. *Controlling for high-risk, age, and sex*			
*k*1	0.52	0.82	0.413
*k*2	**0.23**	**3.62**	**<0.001**
*k*3	0.01	0.22	0.827
High risk	**0.18**	**4.47**	**<0.001**
Age at last interview/W6	**0.01**	**10.00**	**<0.001**
Sex	**0.10**	**2.44**	**0.015**

*k*1 = Cancer + Other Cardiovascular + Hypothyroidism + Stroke + Diabetes + Hyper-thyroid + Convulsions + Gallbladder disease; *k*2 = Head injury; *k*3 = Hypertension.

Bold values are associated with significant findings.

#### Structural equation models

Medical disorders were classified into three broad categories, (1) lipid-associated medical disorders (LAMD), a hypothetical construct variable representing chronic lipid disorders (diabetes, gallbladder disease, hypertension, and other cardiovascular diseases) was compared to two other constructs that consisted of medical disorders which are hypothesized to be independent or moderately associated with lipid dyshomeostasis: (2) lipid-independent medical disorders (LIMD; cancer), and (3) intermediately associated medical disorders (IAMD; convulsion, head injury, stroke) (Table 3, Supplemental Table 1). In order to determine the association of medical disorders with depression as an outcome, we used structural equation models (SEM) [42] to investigate a priori hypotheses on relationships between observed and latent variables. Based on longitudinal data from the multigenerational familial risk study, we examined the association between lifetime MDD as an outcome and the three medical disorder categories (LAMD, LIMD, IAMD) while controlling for participant’s age at the last interview (wave 6; W6) sex, and familial risk status by applying structural equation models in the R software (version 3.6.1) and package “lavaan”. For analyses of the cognition scores in a subset of participants (*N* = 150) collected at W6, structural equation models were applied to WASI [43]: Similarities *T*-scores and Verbal IQ sum of *T*-scores as the outcome to separately investigate their association with three medical disorder categories, controlling for age at W6, sex, and familial risk status as described above.

**Table 3:** Structural equation models with lifetime MDD and cognition as outcome.

Predictors	Outcome: Lifetime MDD					
	Sample: G2 + G3, *N* = 526			Sample: High risk, G2 + G3, *N* = 354		
	Estimate	*z*-value	*p*(>|*z*|)	Estimate	*z*-value	*p*(>|*z*|)
1. *Basic model*						
LAMD (Lipid-Associated Medical Disorder)	1.37	4.08	**<0.001**	1.50	3.83	**<0.001**
LIMD (Lipid-Independent Medical Disorder)	0.00	0.03	0.976	−0.03	−0.19	0.848
IAMD (Intermediately Associated Medical Disorder)	2.01	0.96	0.339	1.35	0.65	0.519
2. *Controlling for familial risk, age, and sex*						
LAMD	0.37	1.76	0.078	0.52	1.98	**0.048**
LIMD	−0.04	−0.41	0.680	−0.06	−0.54	0.586
IAMD	2.98	1.77	0.078	2.91	1.27	0.204
High risk	0.19	4.59	**<0.001**			
Age at last interview/W6	0.01	9.76	**<0.001**	0.01	9.83	**<0.001**
Sex	0.10	2.45	**0.014**	0.05	1.11	0.266

Predictors	Outcome: Similarities subtest T-scores			Outcome: Verbal IQ sum of T-scores		
	Sample: G2 + G3, *N* = 150					
	Estimate	*z*-value	*p*(>|*z*|)	Estimate	*z*-value	*p*(>|*z*|)
1. *Basic model*						
LAMD	16.79	1.83	0.068	22.61	1.73	0.084
LIMD	−1.70	−0.58	0.564	−2.70	−0.52	0.605
IAMD	−28.37	−1.75	0.081	−42.96	−1.52	0.128
2. *Controlling for familial risk, age, and sex*						
LAMD	25.80	2.12	**0.034**	39.97	2.05	**0.040**
LIMD	−4.60	−1.38	0.169	−8.83	−1.43	0.154
IAMD	−31.56	−1.83	0.067	−52.30	−1.66	0.098
High risk	4.19	2.92	0.003	8.66	3.34	0.001
Age at last interview/W6	0.08	1.67	0.095	0.14	1.62	0.105
Sex	2.01	1.42	0.155	2.22	0.87	0.384

Bold values are associated with significant findings.

#### Survival analysis

**Fig. 2:**
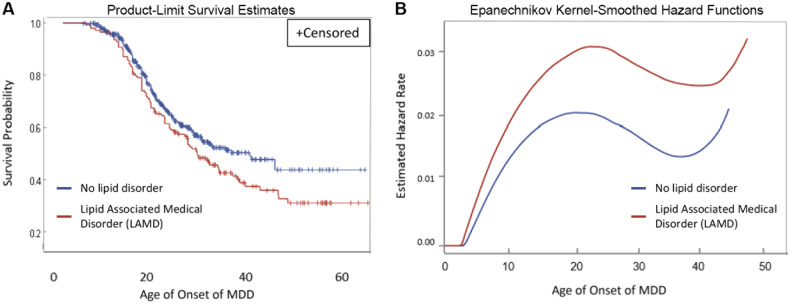
Subjects with lipid-associated medical disorders (LAMD, red) display reduced survival probability and enhanced hazard rate compared to those with no lipid disorder (blue). Survival analysis—age of onset of depression stratified by lifelong incidence of lipid disorder using a non-parametric statistical LIFETEST procedure in the SAS software (version 9.4) to estimate **(A)** the survival probabilities of lifetime MDD stratified by LAMD, and **(B)** the age-specific hazard rates of MDD in 10-year intervals stratified by LAMD.

In order to determine if the lifetime incidence of LAMD affected the cumulative incidence of depression, we conducted a survival analysis. The longitudinal nature of the multigenerational familial risk study allowed us to conduct a survival analysis of the cumulative incidence of depression, comparing individuals who had an LAMD during their lifetime with those who did not. We conducted survival analysis [44] using the non-parametric LIFETEST procedure in SAS (version 9.4) to estimate the survival probabilities of lifetime MDD stratified by LAMD (Fig. 2A) and the age-specific hazard rates of MDD in 10-year intervals stratified by LAMD (Fig. 2B).

### NHANES study

#### Population

This study used the combined data from the two NHANES cycles (2011–2012, 2013–2014). The specific sampling strategy has been published [45]. After meeting the study criteria, a total of 2377 participants were included in the analysis (Supplemental Table 2, Supplemental Fig. 1).

#### Multivariate analysis

Multivariate linear regression models were conducted in SAS on Demand to analyze the association between HDL and Cholesterol–HDL ratio (as continuous and binary variables, respectively). Models were adjusted for age, sex, depression, and body mass index (BMI) as covariates. Estimated effect size (*β*) was reported, and statistical significance was set at *α* = 0.05. To determine how the presence or absence of depression might influence the indirect pathway of the relationship between HDL levels and cognitive performance, we conducted a mediation analysis. To determine if depression mediates the interaction between HDL and cognitive *z*-scores, a multivariate linear regression model for HDL and cognitive scores was performed after stratifying by depression status. We also conducted multivariate mediation analysis for estimating total, direct, indirect, and controlled effects using SAS [(Procedure Causal Mediation (proc causal med)] within the same model.

#### NHANES study assessments

The National Center for Health Statistics of the Centers for Disease Control and Prevention (CDC) is a continuous cross-sectional survey to represent the US population of non-institutionalized civilians that conducts in-home interviews and physical examinations at mobile examination centers [46].

Depressive symptoms were measured using the Patient Health Questionnaire-9 (PHQ-9) [47]. The TC to HDL ratio was generated and is now increasingly utilized as a useful predictor of cardiovascular heart disease (CHD) risk compared to LDL or TC alone [48, 49]. For the TC to HDL ratio, a cut of 3.5 suggested by the American Heart Association was used to create a binary variable [49]. Cognitive performance was obtained through the Consortium to Establish a Registry for Alzheimer’s Disease (CERAD) [50]. Word Learning sub-tests, including delayed word recall tests (DWRT), Animal Fluency test (AFT), and the Digit Symbol Substitution test (DSST) as previously published [46]. The cognitive test scores were converted to *z*-scores using sample mean and standard deviation.

## Results

### Familial risk study

A total of 526 individuals from the multigenerational familial risk study, 273 (51.9%) from G2 and 253 (48.1%) from G3, were included. Mean age was 32.5 (±16.1) years, 279 (53%) were females, and 354 (67.3%) were at familial high risk for depression. Demographic, health, and family history characteristics are summarized in Table 1, including the subset of individuals who had cognitive testing, including the WASI [43] at W6.

We aimed to investigate the association between lifetime LAMD and lifetime depression in a longitudinal cohort. We hypothesized that depression is associated with lipid dyshomeostasis, which underlies multiple medical conditions, including diabetes, gallbladder disease, hypertension, and other cardiovascular diseases based on disease etiology. We identified medical conditions associated with lipid dyshomeostasis that clustered in an unsupervised model. The results of the cluster analysis show that the *k*1 predictor cluster (cancer, other cardiovascular, hypo- and hyperthyroidism, stroke, diabetes, convulsions, and gallbladder disease) is significantly associated with MDD in the basic model (*β* = 2.56, *z*-value = 2.91, *p* = 0.004) (Table 2, Fig. 1). In the second model, which included covariates familial risk status, age, and sex, the effect of *k*1 became insignificant, but *k*2 (head injury) was a significant predictor of MDD (*k*2: *β* = 0.23, *z*-value = 3.62, *p*-value < 0.001).

To complement the cluster analysis, we constructed a hypothetical composite construct variable for LAMD and two control hypothetical construct variables for medical conditions with etiologies LIMD or IAMD on lipid dyshomeostasis, based on relevance to LAMD. Table 3 shows the findings of the structural equation models for the main sample (*N* = 526) with lifetime MDD as the outcome. The analysis was performed in the basic model and in a controlled model. Individually, LAMD was not associated with MDD after adjusting for familial risk status, age, and sex in a logistic regression model (Supplemental Table 1). However, the composite variable for LAMD was significantly associated with MDD while lipid-independent and intermediately associated conditions were not (Table 3). In the basic model, the LAMD composite variable was positively associated with lifetime MDD (*β* = 1.37, *p* < 0.001), although it did not maintain significance in the controlled model (*β* = 0.37, *p* = 0.078). However, among individuals with high familial risk for depression (*N* = 354), the structural equation models with lifetime MDD as the outcome show that LAMD is a significant predictor of MDD in the basic model (*β* = 1.50, *p* < 0.001) and after controlling for familial risk, age, and sex (*β* = 0.52, *p* = 0.048) (Table 3). Additionally, age was a significant predictor of MDD (*β* = 0.01, *p* < 0.001), suggesting that age is an important factor to consider when identifying individuals at higher risk for depression. However, sex was not a significant predictor of MDD in the high-risk sample. Interestingly, the hypothesized composite variables LIMD and IAMD were not significant predictors of MDD, suggesting that LAMD is unique among co-morbid medical conditions for predicting depression (Table 3).

Survival curves show the cumulative probability of surviving depression from age 0 to the age of MDD onset (Fig. 2). The *Y*-axis is the probability of being lifetime non-depressed, and the *X*-axis is the age of onset of MDD. The red line on the graph shows that individuals who have LAMD, are more likely to develop depression (censor). This indicates that the lifetime occurrence of LAMD is associated with an increased risk of developing depression. This finding is recapitulated in the hazard function for LAMD (Fig. 2B), in which the instantaneous risk of depression is greater in the LAMD group. Two groups of depression-onset individuals were compared, i.e., those with depression onset before lipid disorder onset and those with depression onset after onset of lipid disorder. There was no difference in the two groups suggesting that the sequence of occurrence of the two disorders is independent of the survival probability of depression (data not shown).

We used a subset of the sample (*N* = 150) for exploratory analysis of the association of LAMD with cognition at W6. In the adjusted model, LAMD was positively associated with two verbal outcomes from the WASI [43]: Similarities subtest *T*-scores (*β* = 25.8, *p* = 0.034) and Verbal IQ sum of *T*-scores (*β* = 39.97, *p* = 0.040) (Table 3). We found through specific analysis that lifetime MDD did not mediate the effect of LAMD on cognition (data not shown). However, we cannot be certain that our sample size was sufficient to detect a mediation effect.

### NHANES Study

A total of 2377 individuals with a mean age of 69 (±7) years, 1207 (54%) females, and 1105 (59%) high values of HDL (>40 mg/dL) were included. All the demographic characteristics are presented in Supplemental Table 2 and exclusion criteria for this study are presented in Supplemental Fig. 1. The results of the multivariate linear regression model of lipid parameters predicted by cognitive *z*-scores in an unadjusted (Model 1) and a model adjusted model for age, sex, and body mass index (BMI) (Model 2) are shown in Supplemental Table 3. We observed that higher levels of HDL were associated with higher digit symbol substitution test (DSST) *z*-scores (*β* = 0.006, SE = 0.001, *p* < 0.001), Animal Fluency test (AFT) *z*-scores (*β* = 0.007, SE = 0.001, *p* < 0.001) and Delayed Word Recall test (DWRT) *z*-scores (*β* = 0.004, SE = 0.001, *p* < 0.001) in the adjusted model. In both the unadjusted and adjusted models, high HDL was associated with higher cognitive *z*-scores in all domains, suggesting a protective effect of HDL on cognitive status. In the unadjusted model, a higher Cholesterol/HDL ratio was associated with a lower DSST cognitive z-score. In the adjusted model, the Cholesterol/HDL ratio remained negatively associated with DSST z-scores (*β* = −0.046, SE = 0.016, *p* = 0.004).

**Fig. 3:**
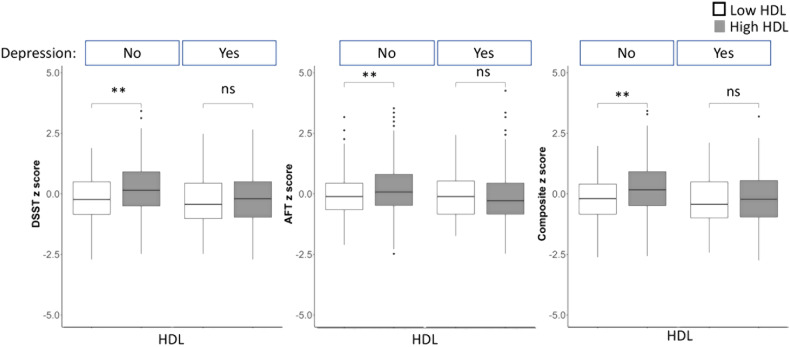
Digital symbol substitution test (DSST), animal fluency test (AFT), and composite cognitive test have higher standardized *z*-scores with higher HDL levels among those without depression but not in those with depression. The boxes contain the 25th–75th percentile values and the solid black lines show the median. The whiskers mark the 5th and 95th percentiles. Outliers are excluded. Significance is indicated by (*) at *p* < 0.05.

Multivariate linear regression analysis examining the relationship between HDL levels and cognitive test scores, stratified by depression status, are shown in Fig. 3 and Supplemental Table 4. In the adjusted model (Model 2), there was a statistically significant positive relationship between HDL levels and AFT *z*-scores only in the group without depression (Depression “No”: *β* = 0.220, SE = 0.063, *p* < 0.001 vs. Depression “Yes”: *β* = −0.049, SE = 0.103, *p* = 0.629). For the DWRT test, there was a significant relationship between HDL levels and cognitive scores in those without depression however, the significance was lost in the adjusted model. Finally, for DSST scores, there was a statistically significant positive relationship between higher HDL levels and cognitive test *z*-scores for the group without depression (*β* = 0.299, SE = 0.060, *p* < 0.001), but not for the group with depression, (*β* = 0.054, SE = 0.102, *p* = 0.598) in the adjusted model suggesting that depression mediated the effect of HDL levels on cognitive scores.

We performed mediation analysis to examine the relationship between HDL levels and cognitive test scores to identify if depression status is a mediator in the pathway between HDL and cognitive measures. We observed that HDL levels are positively associated with cognitive *z*-scores, evidenced by significant total effects across all measurements (Supplemental Table 5; *p* < 0.001 for DSST, AFT, and DWRT in HDL [mg/dL]). This effect persisted in the adjusted model. The analysis revealed a significant natural indirect effect of depression on all cognitive measures (*β* = 0.0005, *p* = 0.037 for DSST; *β* = 0.0003, *p* = 0.049 for AFT, and *β* = 0.0003, *p* = 0.049 for DWRT on HDL [mg/dL]), suggesting that part of HDL’s influence on cognition may be mediated by the presence of depressive symptoms. These results are strengthened by adjusting for demographic and health variables, such as age, sex, and BMI, indicating that depression is a potential mediator of the relationship between HDL and cognitive z-scores (Fig. 2 and Supplemental Table 5).

## Discussion

Our analysis of the multigenerational familial risk for depression study showed that lifetime major depressive disorder (MDD) and verbal cognitive scores were significantly associated with the hypothesized composite variable scores representing lipid-associated medical disorder (LAMD, Table 3). Because the familial risk study is a longitudinal study and deeply phenotyped, we can assess the lifetime incidence of depression and LAMD. We tested the hypothesis that the composite variable representing the combined LAMD was associated with lifetime MDD using log-rank tests. Additionally, using survival analysis, we found that over time, individuals with LAMD are more likely to develop depression than the controls at the same age, since cumulative probability of survival is less in the LAMD group compared to the nonLAMD group (Fig. 2A). These findings are consistent with the hazard function for LAMD (Fig. 2B), in which we found that the instantaneous risk of depression is greater in the LAMD group. We found that lifetime MDD was associated with LAMD in the adjusted model only in the subgroup with a high risk for depression but not in the full sample (Table 3). This result may suggest that the high-risk subgroup, which is known to have a genetic predisposition to depression [33–37, 51], also may have common genetic drivers for LAMD.

Having established the role of lipid dysregulation in depression, we wanted to evaluate the effect of chronic lipid dyshomeostasis on cognition in the LAMD groups. Since the subsample size with cognition scores does not reach the minimum (*N* = 200) required for a structural equation model, we performed an exploratory cross-sectional analysis for the association between LAMD and cognition at wave 6 (Table 3). We found a significant association between LAMD and cognitive scores in the verbal domain.

Based on this exploratory analysis, we conducted a higherpower study in NHANES, resulting in similar findings. Although the studies vary in time, site demographics, and sample size, both have assessed metrics that can be used to address our current hypotheses regarding depression status, lipid dyshomeostasis, and cognition. This enabled us to determine the consistency of results in broader and independent samples, which strengthens the validity of our findings through replicability across diverse sample sets and a range of populations. The limitations of the familial risk study are tempered by the larger sample size and availability of clinical lipid measures in NHANES. However, the NHANES study is, limited by the cross-sectional design, thereby benefitting from the deeply phenotyped longitudinal study of familial risk for depression.

Based on our findings in the familial risk study regarding the association of LAMD, depression, and cognition, we interrogated NHANES, which reports clinical measures of peripheral lipids and also has more power to determine if depression is moderating the effect of lipid dyshomeostasis on cognition. Our analysis of the NHANES found that depression status mediates the effect of HDL levels on cognition (Fig. 3, Supplemental Tables 4 and 5).

Plasma HDL has a well-established protective role in cardiovascular disease. Recent studies strongly indicate that LDL is associated with cognitive impairment, while HDL improves memory [52, 53]. To our knowledge, this study is the first to find that HDL is associated with increased cognitive scores, and this association is only found in people who were not depressed, suggesting mediation of the protective effect of HDL by depression. Further, the Cholesterol/HDL ratio, which is a predictor for poor metabolic health, has been shown in our study to be negatively correlated with cognition scores. These results suggest a positive effect of “good” cholesterol on cognitive scores only in those who are not depressed. When stratified by depression status, the effect of HDL level on cognition was not seen in the group with depression, suggesting that depression status is a mediator that impacts the association between lipid levels and cognition. Although the overall effect (*β*) of the mediating effect of depression on the association of HDL with cognitive score is small and may not represent a clinically meaningful change in diagnosis or disease progression, these studies serve to identify the mechanistic pathway between lipid dyshomeostasis, depression, and cognition. This mediation effect may ultimately hold promise clinically, suggesting that earlier detection of mental health status as well as peripheral lipid dyshomeostasis which are pharmacologically tractable, may ultimately impact cognition.

The significant association of the composite LAMD with MDD and cognitive scores suggests that a chronic state of lipid dyshomeostasis, common to these medical conditions, underlies changes in cognition, especially in the verbal domain. This is consistent with alterations in circulating lipid concentrations which may be linked to pathophysiological pathways related to depression [54]. Further, alterations in lipid metabolism may represent a consequence of depressive symptoms in which patients with depression are more likely to engage in unhealthy behaviors, such as sedentariness and poor nutrition, which may lead to dyslipidemia resulting in metabolic syndrome [12, 55–57]. Mental illness-related distress has been known to impact neurocognitive decline biologically through vascular changes in memory and excessive release of corticosteroids, both causing damage to brain areas associated with memory and learning [58]. Through indirect psychological mechanisms, depressive symptoms are also known to cause deficits in information processing speed and executive functions [59]. The mediating impact of depression further highlights the role of indirect biological and psychological pathways through which depression may aggravate cognitive decline or reduce the benefits of protective lipoprotein.

Our work refines the understanding from previous NHANES analyses which report contrasting results. Previous analyses of the NHANES have reported that cognitive deficits associated with depression are synergistic with diabetes [29]. However, clinical lipid levels have not directly been reported to be associated with cognition in the NHANES, supporting the novelty of our findings. Recently, Lee et al. (2021) also determined the association between lipid parameters and cognition in the NHANES, however, only one cognitive domain was included, and Cholesterol/HDL ratio was not included as a predictor [60]. Additionally, Cepeda et al. (2020), reported that low levels of TC in NHANES (2009-2015) were not associated with an increased risk of depression [11] in contrast to several other studies which report an inverse relationship between depression and levels of TC [12–14]. One difference in our study is that we stratified depression status by a PHQ-9 score of 5 while the previous work in NHANES used a PHQ-9 score of equal or greater than 10 to define depression. Our study may be more sensitive to changes in a non-depressed cohort compared to these previous studies, which are inclusive of those with higher PHQ-9 scores indicative of mild to moderate depressive symptoms.

Lipid dyshomeostasis is known to be an underlying mechanism for vascular dementia as well as Alzheimer’s disease (AD), as previous studies have shown lipid dysregulation to promote AD through both genetic and metabolic pathways [52]. However, the direct effect on cognition has yet to be determined in the context of these medical conditions. Our findings are corroborated by recent work that shows that the major genetic risk factor for late-onset AD involved in cholesterol trafficking, apolipoprotein Eε4, alters cholesterol levels in the brain and may underlie cognitive deficits associated with the disease [61–63]. Additional longitudinal studies regarding cognitive decline and clinical measures of lipid dyshomeostasis are needed to determine the nature of the interaction between lipid dyshomeostasis and depression in shaping cognitive outcomes. Finally, our findings suggest that multimorbidity might be an important variable in understanding depression and also may have value for predicting those at higher risk for depression availing earlier interventions [64]. Moreover, identification of this interaction will lead to future studies to determine if changes in cognition are the result of dietary and lifestyle changes due to depressive symptoms or, alternatively, if yet unknown genetic factors, common to chronic lipid dyshomeostasis in LAMD, drive alterations in brain lipid content and neuronal functioning, ultimately leading to cognitive changes.

### Supplementary information

Supplemental Material

## Data Availability

The familial risk for depression study is longitudinal and began in 1982 pre-dating current procedures for data sharing and therefore, consent for data sharing was not obtained. At the last wave of data collection in 2020, consent for data sharing to be placed in a repository was obtained from a subset of participants who were available in 2020 and who signed consent. This represents a partial sample. These data will be available after 2024 when the data will be accepted to the repository at the NIMH Data Archive (NDA) (https://nda.nih.gov/). We do not have data sharing consent for any other data to be shared and the repository will have only a subset of the data reported in this paper. Data Availability Statement for NHANES. Data can be accessed and downloaded from “NHANES” database (https://www.cdc.gov/nchs/nhanes/index.htm).

## References

[1] Hakim A (2022). Perspectives on the complex links between depression and dementia. Front Aging Neurosci.

[2] Parekh A, Smeeth D, Milner Y, Thure S (2017). The role of lipid biomarkers in major depression. Healthcare (Basel).

[3] Terao T, Iwata N, Kanazawa K, Takano T, Takahashi N, Hayashi T (2000). Low serum cholesterol levels and depressive state in human dock visitors. Acta Psychiatr Scand.

[4] Horsten M, Wamala SP, Vingerhoets A, Orth-Gomer K (1997). Depressive symptoms, social support, and lipid profile in healthy middle-aged women. Psychosom Med.

[5] Rabe-Jablonska J, Poprawska I (2000). Levels of serum total cholesterol and LDL-cholesterol in patients with major depression in acute period and remission. Med Sci Monit.

[6] Olusi SO, Fido AA (1996). Serum lipid concentrations in patients with major depressive disorder. Biol Psychiatry.

[7] Lehto SM, Niskanen L, Tolmunen T, Hintikka J, Viinamaki H, Heiskanen T (2010). Low serum HDL-cholesterol levels are associated with long symptom duration in patients with major depressive disorder. Psychiatry Clin Neurosci.

[8] de Freitas JA, Lima LM, Ranieri JL, Olivieri JC, Fragoso HJ, Chinzon D (2002). [Evaluation of efficacy, safety and tolerability rabeprazole in treatment of acid-peptic diseases]. Arq Gastroenterol.

[9] Sadeghi M, Roohafza H, Afshar H, Rajabi F, Ramzani M, Shemirani H (2011). Relationship between depression and apolipoproteins A and B: a case-control study. Clinics (Sao Paulo).

[10] Lin Z, Lawrence WR, Huang Y, Lin Q, Gao Y (2021). Classifying depression using blood biomarkers: a large population study. J Psychiatr Res.

[11] Cepeda MS, Kern DM, Blacketer C, Drevets WC (2020). Low levels of cholesterol and the cholesterol type are not associated with depression: results of a cross-sectional NHANES study. J Clin Lipido.

[12] Shin JY, Suls J, Martin R (2008). Are cholesterol and depression inversely related? A meta-analysis of the association between two cardiac risk factors. Ann Behav Med.

[13] Morgan RE, Palinkas LA, Barrett-Connor EL, Wingard DL (1993). Plasma cholesterol and depressive symptoms in older men. Lancet.

[14] Sampson M, Ling C, Sun Q, Harb R, Ashmaig M, Warnick R (2020). A new equation for calculation of low-density lipoprotein cholesterol in patients with normolipidemia and/or hypertriglyceridemia. JAMA Cardiol.

[15] Jia QF, Yang HX, Zhuang NN, Yin XY, Zhu ZH, Yuan Y (2020). The role of lipoprotein profile in depression and cognitive performance: a network analysis. Sci Rep.

[16] Qi X, Wang S, Huang Q, Chen X, Qiu L, Ouyang K (2024). The association between non-high-density lipoprotein cholesterol to high-density lipoprotein cholesterol ratio (NHHR) and risk of depression among US adults: a cross-sectional NHANES study. J Affect Disord.

[17] Zhang L, Yin J, Sun H, Yang J, Liu Y (2023). Association between atherogenic coefficient and depression in US adults: a cross-sectional study with data from National Health and Nutrition Examination Survey 2005–2018. BMJ Open.

[18] Zhu X, Zhao Y, Li L, Liu J, Huang Q, Wang S (2023). Association of non-HDL-C and depression: a cross-sectional analysis of the NHANES data. Front Psychiatry.

[19] Chew H, Solomon VA, Fonteh AN (2020). Involvement of lipids in Alzheimer’s disease pathology and potential therapies. Front Physiol.

[20] Wang Q, Zang F, He C, Zhang Z, Xie C, Alzheimer’s Disease Neuroimaging I (2022). Dyslipidemia induced large-scale network connectivity abnormality facilitates cognitive decline in the Alzheimer’s disease. J Transl Med.

[21] Ma YH, Shen XN, Xu W, Huang YY, Li HQ, Tan L (2020). A panel of blood lipids associated with cognitive performance, brain atrophy, and Alzheimer’s diagnosis: a longitudinal study of elders without dementia. Alzheimers Dement (Amst).

[22] Erb SJ, Schappi JM, Rasenick MM (2016). Antidepressants accumulate in lipid rafts independent of monoamine transporters to modulate redistribution of the G protein, galphas. J Biol Chem.

[23] Anacker C, Zunszain PA, Cattaneo A, Carvalho LA, Garabedian MJ, Thuret S (2011). Antidepressants increase human hippocampal neurogenesis by activating the glucocorticoid receptor. Mol Psychiatry.

[24] Malberg JE, Eisch AJ, Nestler EJ, Duman RS (2000). Chronic antidepressant treatment increases neurogenesis in adult rat hippocampus. J Neurosci.

[25] Casarotto PC, Girych M, Fred SM, Kovaleva V, Moliner R, Enkavi G (2021). Antidepressant drugs act by directly binding to TRKB neurotrophin receptors. Cell.

[26] Glomset JA (2006). Role of docosahexaenoic acid in neuronal plasma membranes. Sci STKE.

[27] Zhang SF, Chen HM, Xiong JN, Liu J, Xiong J, Xie JZ (2022). Comparison of cognitive impairments with lipid profiles and inflammatory biomarkers in unipolar and bipolar depression. J Psychiatr Res.

[28] Shao TN, Yin GZ, Yin XL, Wu JQ, Du XD, Zhu HL (2017). Elevated triglyceride levels are associated with cognitive impairments among patients with major depressive disorder. Compr Psychiatry.

[29] Wei J, Ying M, Xie L, Chandrasekar EK, Lu H, Wang T (2019). Late-life depression and cognitive function among older adults in the U.S.: The National Health and Nutrition Examination Survey, 2011–2014. J Psychiatr Res.

[30] Musunuru K, Kathiresan S (2016). Surprises from genetic analyses of lipid risk factors for atherosclerosis. Circ Res.

[31] Musunuru K, Kathiresan S (2019). Genetics of common, complex coronary artery disease. Cell.

[32] Yoon H, Shaw JL, Haigis MC, Greka A (2021). Lipid metabolism in sickness and in health: emerging regulators of lipotoxicity. Mol Cell.

[33] Weissman MM, Gammon GD, John K, Merikangas KR, Warner V, Prusoff BA (1987). Children of depressed parents. Increased psychopathology and early onset of major depression. Arch Gen Psychiatry.

[34] Weissman MM, Wickramaratne P, Nomura Y, Warner V, Verdeli H, Pilowsky DJ (2005). Families at high and low risk for depression: a 3-generation study. Arch Gen Psychiatry.

[35] Weissman MM, Wickramaratne P, Nomura Y, Warner V, Pilowsky D, Verdeli H (2006). Offspring of depressed parents: 20 years later. Am J Psychiatry.

[36] Weissman MM, Warner V, Wickramaratne P, Moreau D, Olfson M (1997). Offspring of depressed parents. 10 years later. Arch Gen Psychiatry.

[37] Weissman MM, Talati A, Gameroff MJ, Pan L, Skipper J, Posner JE (2021). Enduring problems in the offspring of depressed parents followed up to 38 years. EClinicalMedicine.

[38] Kramer RA, Warner V, Olfson M, Ebanks CM, Chaput F, Weissman MM (1998). General medical problems among the offspring of depressed parents: a 10-year follow-up. J Am Acad Child Adolesc Psychiatry.

[39] Mannuzza S, Fyer AJ, Klein DF, Endicott J (1986). Schedule for Affective Disorders and Schizophrenia-Lifetime Version modified for the study of anxiety disorders (SADS-LA): rationale and conceptual development. J Psychiatr Res.

[40] Orvaschel H, Puig-Antich J, Chambers W, Tabrizi MA, Johnson R (1982). Retrospective assessment of prepubertal major depression with the Kiddie-SADS-e. J Am Acad Child Psychiatry.

[41] Cullen B, Gameroff MJ, Ward J, Bailey MES, Lyall DM, Lyall LM (2023). Cognitive Function in People With Familial Risk of Depression. JAMA Psychiatry.

[42] Streiner DL (2006). Building a better model: an introduction to structural equation modelling. Can J Psychiatry.

[43] Wechsler D. Wechsler Abbreviated Scale of Intelligence. The Psychological Corporation: Harcourt Brace & Company. New York, NY. 1999.

[44] Cox DRO, D. Analysis of survival data. New York: Chapman and Hall/CRC;1984.

[45] Johnson CL, Paulose-Ram R, Ogden CL, Carroll MD, Kruszon-Moran D, Dohrmann SM, et al. National health and nutrition examination survey: analytic guidelines, 1999–2010. Vital Health Stat 2013;2:1–24. 25090154

[46] National Health and Nutrition Examination Survey (NHANES). 2023. https://www.cdc.gov/nchs/nhanes/index.htm.

[47] Kroenke K, Spitzer RL, Williams JB (2001). The PHQ-9: validity of a brief depression severity measure. J Gen Intern Med.

[48] Castelli WP (1984). Epidemiology of coronary heart disease: the Framingham study. Am J Med.

[49] Ingelsson E, Schaefer EJ, Contois JH, McNamara JR, Sullivan L, Keyes MJ (2007). Clinical utility of different lipid measures for prediction of coronary heart disease in men and women. JAMA.

[50] Fillenbaum GG, van Belle G, Morris JC, Mohs RC, Mirra SS, Davis PC (2008). Consortium to establish a registry for Alzheimer’s disease (CERAD): the first twenty years. Alzheimers Dement.

[51] Weissman MM, Merikangas KR, Wickramaratne P, Kidd KK, Prusoff BA, Leckman JF (1986). Understanding the clinical heterogeneity of major depression using family data. Arch Gen Psychiatry.

[52] Hottman DA, Chernick D, Cheng S, Wang Z, Li L (2014). HDL and cognition in neurodegenerative disorders. Neurobiol Dis.

[53] Schultz BG, Patten DK, Berlau DJ (2018). The role of statins in both cognitive impairment and protection against dementia: a tale of two mechanisms. Transl Neurodegener.

[54] Pan A, Keum N, Okereke OI, Sun Q, Kivimaki M, Rubin RR (2012). Bidirectional association between depression and metabolic syndrome: a systematic review and meta-analysis of epidemiological studies. Diabetes Care.

[55] Persons JE, Fiedorowicz JG (2016). Depression and serum low-density lipoprotein: a systematic review and meta-analysis. J Affect Disord.

[56] Lin PY, Huang SY, Su KP (2010). A meta-analytic review of polyunsaturated fatty acid compositions in patients with depression. Biol Psychiatry.

[57] Mensink RP, Zock PL, Kester AD, Katan MB (2003). Effects of dietary fatty acids and carbohydrates on the ratio of serum total to HDL cholesterol and on serum lipids and apolipoproteins: a meta-analysis of 60 controlled trials. Am J Clin Nutr.

[58] Korczyn AD, Halperin I (2009). Depression and dementia. J Neurol Sci.

[59] Steffens DC, Potter GG (2008). Geriatric depression and cognitive impairment. Psychol Med.

[60] Lee J, Lee S, Min JY, Min KB (2021). Association between serum lipid parameters and cognitive performance in older adults. J Clin Med.

[61] Blanchard JW, Akay LA, Davila-Velderrain J, von Maydell D, Mathys H, Davidson SM (2022). APOE4 impairs myelination via cholesterol dysregulation in oligodendrocytes. Nature.

[62] Dolgin E (2022). This is how an Alzheimer’s gene ravages the brain. Nature.

[63] Carlstrom K, Castelo-Branco G (2022). Alzheimer’s risk variant APOE4 linked to myelin-assembly malfunction. Nature.

[64] Read JR, Sharpe L, Modini M, Dear BF (2017). Multimorbidity and depression: a systematic review and meta-analysis. J Affect Disord.

