# Supplementary material for: Depression and cognition are associated with lipid dysregulation in both a multigenerational study of depression and the National Health and Nutrition Examination Survey
Source: Transl Psychiatry. 2024 Mar 12;14:142. doi: 10.1038/s41398-024-02847-6 (PMC10928164; doi:10.1038/s41398-024-02847-6)
Supplement: Supplementary file 1 — Supplemental Material [file 41398_2024_2847_MOESM1_ESM.docx]

**Supplemental Information:**

**Supplemental Table 1.** Individual sub-scale specific disorders with MDD and Cognition

**Supplemental Table 2:** Characteristics of the participants included in the NHANES (2011-2014) analysis

# Supplemental Table 3: Multivariate Linear Regression Model Lipid panel and cognitive test scores

# Supplemental Table 4: Multivariate linear regression analysis HDL and cognitive test scores Supplemental Table 5: Multivariate mediation analysis in the relationship between HDL levels and cognitive tests stratified by depression.

**Supplemental Figure 1.** NHANES Exclusion criteria flow chart needed for NHANES analysis.

**Supplemental Table 1.** Individual sub-scale specific disorders with MDD and Cognition

| MDD association with each medical disorder - Logistic Regression | | | | | | |
| --- | --- | --- | --- | --- | --- | --- |
|  | **Basic model** | | | **Controlled model for familial risk, age, and sex** | | |
| **Variable** | **Estimate** | **chi-square** | **p-value** | **Estimate** | **chi-square** | **p-value** |
| **LAMD** | 0.51 | 25.63 | **<0.001** | 0.10 | 0.68 | 0.410 |
| Hypertension | 0.46 | 12.22 | **<0.001** | 0.06 | 0.17 | 0.684 |
| Cardiovascular | 0.30 | 3.36 | 0.067 | 0.04 | 0.05 | 0.826 |
| Hyperthyreoidism | 0.35 | 1.42 | 0.233 | 0.02 | 0.00 | 0.956 |
| Hypothyroidism | 0.86 | 13.43 | **<0.001** | 0.48 | 3.38 | 0.066 |
| Gallbladd | 0.83 | 8.31 | **0.004** | 0.25 | 0.68 | 0.410 |
| Diabetes | 0.32 | 2.07 | 0.150 | -0.05 | 0.05 | 0.829 |
| Hypercholesterolemia | 0.53 | 16.54 | **<0.001** | 0.03 | 0.05 | 0.827 |
| **LIMD** (cancer) | 0.52 | 8.52 | **0.004** | 0.09 | 0.19 | 0.663 |
| **IAMD** | 0.46 | 17.54 | **<0.001** | 0.44 | 13.14 | **<0.001** |
| Convulsion | 0.35 | 2.17 | 0.141 | 0.26 | 1.03 | 0.311 |
| Head injury | 0.50 | 11.93 | **0.001** | 0.57 | 12.58 | **<0.001** |
| Stroke | 0.38 | 56.28 | **0.022** | 0.22 | 1.51 | 0.219 |
| Cognition Scale association with each medical disorder - Continuous Logistic Regression | | | | | | |
|  | **Basic model** | | | **Controlled model for familial risk, age, and sex** | | |
| **Outcome WA001042** | **Estimate** | **t-value** | **p-value** | **Estimate** | **t-value** | **p-value** |
| **LAMD** | -4.26 | -2.71 | **0.008** | -7.16 | -4.17 | **<0.001** |
| Hypertension | -2.14 | -1.05 | 0.298 | -3.59 | -1.64 | 0.103 |
| Cardiovascular | -3.40 | -1.25 | 0.211 | -3.68 | -1.36 | 0.175 |
| Hyperthyreoidism | -2.76 | -0.58 | 0.563 | -3.32 | -0.69 | 0.490 |
| Hypothyroidism | -7.45 | -2.80 | **0.006** | -9.89 | -3.62 | **<0.001** |
| Gallbladd | -0.07 | -0.02 | 0.985 | -2.17 | -0.63 | 0.533 |
| Diabetes | -2.71 | -0.79 | 0.428 | -3.59 | -1.04 | 0.298 |
| Hypercholesterolemia | -0.54 | -0.27 | 0.786 | -1.62 | -0.73 | 0.465 |
| **LIMD (cancer)** | 1.27 | 0.50 | 0.619 | 0.73 | 0.27 | 0.785 |
| **IAMD** | -2.63 | -1.36 | 0.176 | -3.02 | -1.58 | 0.117 |
| Convulsion | -13.12 | -2.44 | **0.016** | -13.60 | -2.56 | **0.011** |
| Head injury | -2.62 | -1.21 | 0.227 | -2.87 | -1.34 | 0.183 |
| Stroke | -1.84 | -0.51 | 0.613 | -2.65 | -0.72 | 0.476 |
| **Outcome WA001048** | **Estimate** | **t-value** | **p-value** | **Estimate** | **t-value** | **p-value** |
| **LAMD** | -7.82 | -2.75 | **0.007** | -12.85 | -4.16 | **<0.001** |
| Hypertension | -6.05 | -1.64 | 0.103 | -8.93 | -2.29 | **0.023** |
| Cardiovascular | -5.24 | -1.07 | 0.288 | -5.64 | -1.16 | 0.248 |
| Hyperthyreoidism | -2.38 | -0.28 | 0.782 | -2.50 | -0.29 | **0.023** |
| Hypothyroidism | -12.14 | -2.52 | **0.013** | -15.91 | -3.21 | **0.002** |
| Gallbladd | -4.30 | -0.70 | 0.486 | -8.42 | -1.35 | 0.179 |
| Diabetes | -5.49 | -0.89 | 0.374 | -6.60 | -1.07 | 0.288 |
| Hypercholesterolemia | -2.47 | -0.69 | 0.494 | -4.84 | -1.22 | 0.225 |
| **LIMD** (cancer) | 2.09 | 0.45 | 0.652 | 1.57 | 0.33 | 0.745 |
| **IAMD** | -3.56 | -1.02 | 0.310 | -4.25 | -1.23 | 0.221 |
| Convulsion | -21.41 | -2.20 | **0.030** | -22.16 | -2.31 | **0.022** |
| Head injury | -3.78 | -0.97 | 0.334 | -4.42 | -1.15 | 0.254 |
| Stroke | -1.83 | -0.28 | 0.781 | -2.56 | -0.38 | 0.702 |
| Individual sub-scale specific medical conditions with lifetime MDD as outcome- logistic (binary outcome) regression, Logistic regression for subset specific with all cognition scores as outcomes. | | | | | | |

**Supplemental Table 2:** Characteristics of the participants included in the NHANES (2011-2014) analysis.

|  | | **All** | **HDL (low)**  **≤ 40 mg/dl** | **HDL (high)**  **> 40 mg/dl** | **Cholesterol/HDL (low) ≤ 3.5** | **Cholesterol/HDL (high) > 3.5** |
| --- | --- | --- | --- | --- | --- | --- |
|  | |  |  |  |  |  |
| N | | 2377 | 425 | 1952 | 1143 | 1234 |
| Age (SD) | | 69.4 (7) | 69.0 (7) | 69.5 (7) | 69.8 (7) | 68.4 (7) |
| Gender (%) | |  |  |  |  |  |
| Male | | 1170 (46) | 323 (73) | 847 (41) | 510 (42) | 698 (51) |
| Female | | 1207 (54) | 102 (27) | 1105 (59) | 633 (58) | 613 (49) |
| Race (%) | |  |  |  |  |  |
| Mexican American | | 211 (3) | 46 (4) | 165 (3) | 84 (3) | 127 (4) |
| Other Hispanic | | 248 (4) | 54 (5) | 194 (4) | 82 (3) | 166 (5) |
| Non-Hispanic White | | 1159 (80) | 206 (78) | 953 (80) | 599 (81) | 560 (78) |
| Non-Hispanic Black | | 526 (8) | 78 (7) | 448 (8) | 269 (8) | 257 (7) |
| Other Race- Including Multi-Racial | | 233 (3) | 41 (6) | 192 (5) | 109 (5) | 124 (6) |
| Education (%) | |  |  |  |  |  |
| Less than 9th grade | | 255 (6) | 63 (8) | 192 (5) | 98 (4) | 157 (7) |
| 9-11th grade (incl. 12th with no diploma) | 309 (10) | | 58 (9) | 251 (9) | 131 (8) | 178 (12) |
| High school graduate (GED or equivalent) | 563 (22) | | 113 (25) | 450 (22) | 267 (22) | 296 (22) |
| Some college or AA degree | 674 (31) | | 105 (33) | 569 (32) | 323 (30) | 351 (33) |
| College Graduate or > | 574 (31) | | 85(25) | 489 (32) | 323 (36) | 251 (26) |
| Diabetes Mellitus (%) |  | |  |  |  |  |
| No | 1823 (81) | | 270 (64) | 1553 (84) | 882 (83) | 941 (79) |
| Yes | 554 (19) | | 155 (6) | 399 (16) | 261 (17) | 293 (21) |
| CAD (%) |  | |  |  |  |  |
| No | 2174 (91) | | 363 (83) | 1811 (93) | 1041 (92) | 1133 (90) |
| Yes | 203 (9) | | 62 (17) | 141 (7) | 102 (8) | 101 (10) |
| Hypertension (%) |  | |  |  |  |  |
| No | 941 (38) | | 155 (36) | 786 (45) | 449 (47) | 492 (39) |
| Yes | 1436 (62) | | 270 (64) | 1166 (55) | 694 (53) | 742 (61) |
| Depression (%) |  | |  |  |  |  |
| No | 1774 (78) | | 302 (74) | 1472 (78) | 875 (80) | 899 (75) |
| Yes | 603 (22) | | 123 (26) | 480 (22) | 268 (20) | 335 (25) |
| BMI mean (SD) | 29 (6) | | 31 (5) | 28 (6) | 28 (6) | 30 (6) |
| AFT mean (SD) | 18 (5) | | 17 (5) | 18 (5) | 18 (5) | 18 (5) |
| DSST mean (SD) | 53 (17) | | 48 (16) | 54 (17) | 54 (17) | 52 (17) |
| DWRT mean (SD) | 6 (2) | | 6 (2) | 6 (2) | 6 (2) | 6 (2) |
| Composite Score mean (SD) | 77 (21) | | 72 (20) | 79 (21) | 78 (21) | 76 (21) |
| PHQ mean (SD) | 3 (4) | | 4 (5) | 3 (4) | 2 (4) | 1. (4) |

Data was analyzed by applying interview and MEC weights as suggested by NHANES. SD reported not weight adjusted. AFT: Animal Fluency Test, DWRT: Delayed Word Recall Test, DSST: Digital Symbol Substitution Test, CAD: Coronary Artery Disease.

|  | **Model 1^a^** | | | **Model 2^b^** | |
| --- | --- | --- | --- | --- | --- |
|  | **ꞵ(SE)** | **p-value** | | **ꞵ(SE)** | **p-value** |
|  | **DSST** | | | | |
| HDL | **0.008 (0.002)** | **< 0.001** | | **0.006 (0.001)** | **<0.001** |
| HDL (Low, High) | **0.339 (0.053)** | **< 0.001** | | **0.263 (0.051)** | **<0.001** |
| Cholesterol-HDL-Ratio | -0.031 (0.016) | 0.063 | | **-0.046 (0.016)** | **0.004** |
| Cholesterol-HDL-Ratio (Low, High) | **-0.078 (0.038)** | **0.048** | | **-0.116 (0.037)** | **0.002** |
|  | **AFT** | | | | |
| HDL | **0.006 (0.001)** | **< 0.001** | | **0.007 (0.001)** | **<0.001** |
| HDL (Low, High) | **0.183 (0.057)** | **< 0.001** | | **0.209 (0.057)** | **<0.001** |
| Cholesterol-HDL-Ratio | -0.001 (0.018) | 0.969 | | -0.022 (0.018) | 0.209 |
| Cholesterol-HDL-Ratio (Low, High) | -0.046 (0.043) | 0.278 | | **-0.098 (0.042)** | **0.018** |
|  | **DWRT** | | | | |
| HDL | **0.006 (0.001)** | **< 0.001** | | **0.004 (0.001)** | **<0.001** |
| HDL (Low, High) | **0.161 (0.054)** | **0.003** | | 0.075 (0.052) | 0.145 |
| Cholesterol-HDL-Ratio | -0.002 (0.017) | 0.899 | | -0.017(0.016) | 0.283 |
| Cholesterol-HDL-Ratio (Low, High) | -0.0296 (0.041) | | 0.465 | -0.070 (0.038) | 0.064 |

**Supplemental Table 3:** Multivariate Linear Regression Model Lipid Panel and Cognitive Tests.

DSST: Digit Symbol Substitution Test (z-scores); AFT: Animal Fluency Test (z-scores); DWRT: Delayed World Recall Test (z-scores); ^a^Model 1: Unadjusted; ^b^Model 2: adjusted for age, sex, BMI. Lipid parameters are measured as (mg/dL). HDL: Low ≤ 40 High > 40, Cholesterol-HDL-Ratio: Low ≤3.5High>3.5.

**Supplemental Table 4:** Multivariate linear regression analysis in the relationship between HDL levels and cognitive tests stratified by depression.

| HDL^c^ |  | **Model 1^a^** | | **Model 2^b^** | |
| --- | --- | --- | --- | --- | --- |
|  |  | **ꞵ(SE)** | **p-value** | **ꞵ(SE)** | **p-value** |
|  | **Depression^d^** | **AFT** | | | |
|  | Yes | -0.081 (0.100) | 0.424 | -0.049 (0.103) | 0.629 |
|  | No | **0.185 (0.062)** | **<0.001** | **0.220 (0.063)** | **<0.001** |
|  | **Depression^d^** | **DWRT** | | | |
|  | Yes | 0.106 (0.09) | 0.270 | 0.04 (0.097) | 0.656 |
|  | No | **0.203 (0.064)** | **<0.001** | 0.101 (0.061) | 0.092 |
|  | **Depression^d^** | **DSST** | | | |
|  | Yes | 0.136 (0.104) | 0.183 | 0.054 (0.102) | 0.598 |
|  | No | **0.396 (0.061)** | **<0.001** | **0.299 (0.060)** | **<0.001** |
| DSST: Digit Symbol Substitution Test (z-scores); AFT: Animal Fluency Test (z-scores); DWRT: Delayed World Recall Test (z-scores);  ^a^Model 1: Unadjusted;  ^b^Model 2: adjusted for age, sex, BMI;  ^c^HDL parameter measured as (mg/dL). HDL: Low ≤ 40 High > 40;  ^d^Depression: Yes = PHQ score < 5 and No = PHQ score ≥ 5; Stratified by Depression Status. | | | | | |

**Supplemental Table 5:** Multivariate mediation analysis showing direct and indirect association between cholesterol ratios and cognitive scores, taking depression as a mediator.

|  |  | **DSST** | | | |
| --- | --- | --- | --- | --- | --- |
| **Mediator: Depression^b^** | **HDL^a^** |  |  | **ꞵ** | **p-value** |
|  |  | **mg/dL** | Total Effect | **0.0060** | **<.0001** |
|  |  |  | Controlled Direct Effect | **0.0065** | **<.0001** |
|  |  |  | Natural Direct Effect | **0.0054** | **<.0001** |
|  |  |  | Natural Indirect Effect | **0.0005** | **0.0374** |
|  |  | **Yes/No** | Total Effect | **0.2660** | **<.0001** |
|  |  |  | Controlled Direct Effect | **0.3049** | **<.0001** |
|  |  |  | Natural Direct Effect | **0.2272** | **<.0001** |
|  |  |  | Natural Indirect Effect | **0.0388** | **0.0048** |
|  |  | **AFT** | | | |
|  |  |  |  | **ꞵ** | **p-value** |
|  |  | **mg/dL** | Total Effect | **0.0058** | **<.0001** |
|  |  |  | Controlled Direct Effect | **0.0065** | **<.0001** |
|  |  |  | Natural Direct Effect | **0.0055** | **<.0001** |
|  |  |  | Natural Indirect Effect | **0.0003** | **0.0489** |
|  |  | **Yes/No** | Total Effect | **0.1681** | **0.0019** |
|  |  |  | Controlled Direct Effect | **0.2209** | **0.0004** |
|  |  |  | Natural Direct Effect | **0.1411** | **0.0090** |
|  |  |  | Indirect Direct Effect | **0.0270** | **0.0093** |
|  |  | **DWRT** | | | |
|  |  |  |  | **ꞵ** | **p-value** |
|  |  | **mg/dL** | Total Effect | **0.004** | **0.0014** |
|  |  |  | Controlled Direct Effect | **0.004** | **0.0049** |
|  |  |  | Natural Direct Effect | **0.004** | **0.0030** |
|  |  |  | Natural Indirect Effect | **0.001** | **0.0495** |
|  |  | **Yes/No** | Total Effect | **0.1046** | **0.0400** |
|  |  |  | Controlled Direct Effect | **0.1180** | **0.0458** |
|  |  |  | Natural Direct Effect | 0.0847 | 0.0945 |
|  |  |  | Natural Indirect Effect | **0.0200** | **0.0160** |

DSST: Digit Symbol Substitution Test (z-scores); AFT: Animal Fluency Test (z-scores); DWRT: Delayed World Recall Test (z-scores); ^a^HDL- levels: in (mg/dL), HDL: Low ≤ 40 High > 40; ^b^Depression: No = PHQ score <5 and Yes = PHQ score ≥ 5.


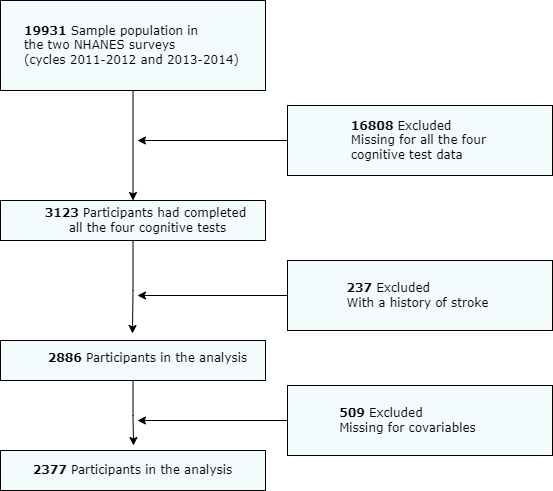


**Supplemental Figure 1.** NHANES exclusion criteria flow chart needed for NHANES analysis.
